# 1163. Influenza Vaccination Coverage through December among Persons Ages Six Months and Older in the Vaccine Safety Datalink, 2017-18 through 2022-23 Influenza Seasons

**DOI:** 10.1093/ofid/ofad500.1003

**Published:** 2023-11-27

**Authors:** Stephanie Irving, Holly C Groom, Edward Belongia, Bradley Crane, Matthew F Daley, Kristin Goddard, Lisa Jackson, Tat’Yana A Kenigsberg, Leslie Kuckler, Allison L Naleway, Suchita A Patel, Hung Fu Tseng, Joshua T B Williams, Eric Weintraub

**Affiliations:** Kaiser Permanente Center for Health Research, Portland, Oregon; Kaiser Permanente Center for Health Research, Portland, Oregon; Marshfield Clinic Research Institute, Marshfield, WI; Kaiser Permanente Center for Health Research, Portland, Oregon; Kaiser Permanente Colorado, Aurora, Colorado; Division of Research Kaiser Permanente Vaccine Study Center, Oakland, California; Kaiser Permanente Washington Health Research Institute, Seattle, WA; Centers for Disease Control and Prevention, Atlanta, Georgia; HealthPartners Institute, Minneapolis, Minnesota; Kaiser Permanente Center for Health Research, Portland, Oregon; Centers for Disease Control and Prevention, Atlanta, Georgia; Kaiser Permanente Southern California, Pasadena, California; Denver Health, Denver, Colorado; Centers for Disease Control and Prevention, Atlanta, Georgia

## Abstract

**Background:**

In the United States, annual vaccination against seasonal influenza is recommended for all people ages ≥ 6 months. Vaccination coverage assessments can identify populations with lower vaccination rates and help to tailor vaccination efforts. Within the Vaccine Safety Datalink population ages ≥ 6 months, we report influenza vaccination coverage through December of the 2022-23 influenza season and compare estimates to similar periods within the 2017-18 through 2021-22 seasons.

**Methods:**

Using electronic health record data, we captured all influenza vaccines administered between August 1 and December 31 of each season. Crude, single-dose coverage was calculated for each season, overall and by sex; age group; race and ethnicity; and number of separate categories of diagnoses associated with an increased risk of severe illness and complications from influenza – these conditions were identified using ICD-10-CM diagnosis codes assigned in the year preceding each influenza season.

**Results:**

Among cohorts of more than 11 million individuals each season, crude influenza vaccination coverage through December ranged from a high of 44% (2020-21) to a low of 37% (2022-23). In each of the six seasons, coverage was lowest among males, 18-49-year-olds (Figure), non-Hispanic Black people, and those without conditions associated with increased risk of severe illness and complications from influenza. While decreases in coverage in recent seasons were present in all age groups, the declines were most dramatic among children: during the 2022-23 season, coverage for children ages < 9 and 9-17 years decreased 25% and 26% (12 and 10 absolute percentage points), respectively, from peak coverage in prior seasons (Figure).

**Figure:**
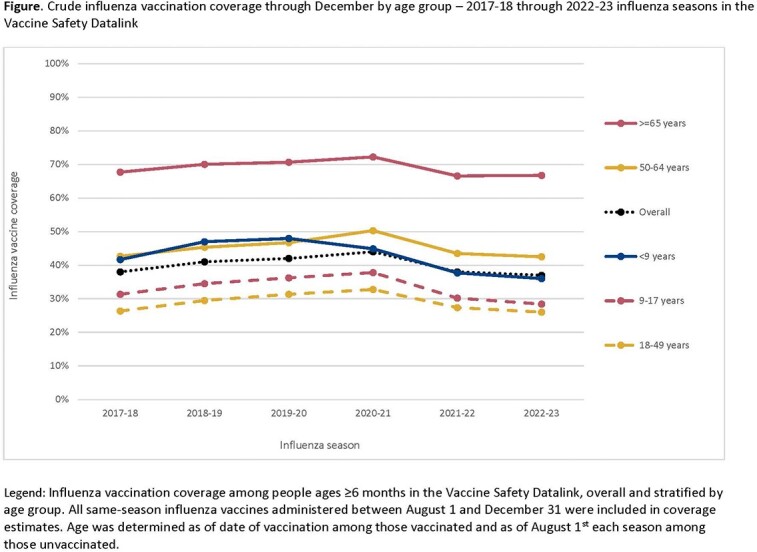

**Conclusion:**

Influenza vaccination coverage increased from the 2017-18 through 2020-21 seasons, then decreased to the lowest level in the 2022-23 season. Overall Vaccine Safety Datalink population estimates are far below United States vaccination targets. We identified persistent disparities in influenza vaccination coverage by sex, age, and race and ethnicity. The overall low coverage, disparities in coverage, and recent decreases in coverage raise significant public health concerns.

**Disclosures:**

**Edward Belongia, MD**, Seqirus: Grant/Research Support **Lisa Jackson, MD, MPH**, Pfizer: Grant/Research Support **Hung Fu Tseng, PhD MPH**, GSK: Grant/Research Support|Moderna: Grant/Research Support

